# Identifying Key Biodiversity Areas Based on Distinct Genetic Diversity

**DOI:** 10.1111/1755-0998.70094

**Published:** 2026-01-10

**Authors:** Sarah Christin Gronefeld, Heriberto López, Robin Schmidt, Axel Hochkirch

**Affiliations:** ^1^ Trier University Department of Biogeography Trier Germany; ^2^ Instituto de Productos Naturales y Agrobiología (IPNA CSIC) San Cristóbal de La Laguna Spain; ^3^ Zoological Institute Technische Universität Braunschweig Braunschweig Germany; ^4^ Musée National D'histoire Naturelle Luxembourg Luxembourg Luxembourg

**Keywords:** conservation, genetic distinctiveness, genetic diversity, genetics structure, KBA, species protection

## Abstract

Key Biodiversity Areas (KBAs) are sites that contribute significantly to the global persistence of biodiversity. Distinct genetic diversity has been introduced as one of the metrics to estimate whether a site holds a threshold proportion of a species' global genetic diversity during the KBA identification process. However, genetic data has so far not been applied in KBA identification due to the lack of thoroughly tested methods and guidance. We tested the suitability of six analytical methods for identification of KBAs based upon genetic data: allelic overlap, Analyses of Molecular Variance (AMOVA), average taxonomic distinctness (AvTD, Δ^+^), effective population size (*N*_e_), the genetic differentiation index (*D*_est_), and the diversity index Simpson's λ. We conclude that Δ^+^, a measure that was developed to measure taxonomic distinctness of biotic communities, performs best in the context of KBA identification as it reflects the unique nature of a species' genetic diversity, is based on simple allele frequencies, and can be easily applied and calculated. AMOVA, *N*_e_, allelic overlap, and our modified version of λ were difficult to apply, interpret, or both. *D*_est_ is easily applied for measuring genetic distinctiveness but not genetic diversity. For this reason, it may not be suitable for prioritising areas for the long‐term protection of the species.

## ABSTRACT (SPANISH)

Las Key Biodiversity Areas (KBAs) son aquellos lugares de importancia mundial que contribuyen de forma significativa a la conservación de la biodiversidad. La diversidad genética diferenciada se ha propuesto como un nuevo parámetro para evaluar si la contribución de un área a la diversidad genética total de una especie es suficiente, según el umbral que determina su reconocimiento como KBA. Sin embargo, este parámetro aún no se ha aplicado debido a la falta de métodos y directrices probados de forma exhaustiva. En este estudio se explora la idoneidad de seis índices o métodos para identificar KBAs usando datos genéticos: superposición alélica, análisis de varianza molecular (AMOVA), diferenciación taxonómica media (AvTD, Δ^+^), tamaño efectivo de la población (*N*_e_), coeficiente de diferenciación genética (*D*_est_) y el índice de diversidad de Simpson (λ). Encontramos que el índice Δ^+^, desarrollado originalmente para medir la diversidad taxonómica de las comunidades bióticas, es el método que mejor funciona para la identificación de KBA, ya que: (i) es el más preciso a la hora de reflejar la naturaleza única de la diversidad genética de una especie, (ii) se basa en frecuencias alélicas y (iii) se puede calcular y aplicar sin problemas. AMOVA, *N*_e_, superposición alélica y nuestra versión modificada de λ resultaron difíciles de aplicar, interpretar o ambas cosas. El índice *D*_est_ se aplica fácilmente, pero sólo mide la diferenciación genética, sin abarcar la diversidad genética, por lo que podría ser inadecuado para priorizar áreas para la protección a largo plazo de las especies.

## 1 Introduction

Global biodiversity policy aims at maintaining biodiversity, but most international or national conservation targets focus on species or ecosystem diversity. The underlying third layer of biodiversity, genetic diversity, is currently insufficiently considered in conservation (Hoban et al. 2021; O'Brien et al. 2022). Genetic variation is being lost at a rapid rate (Leigh et al. 2019) in most species (Shaw et al. 2025) with major consequences for species survival, stability of ecosystems and our society (Des Roches et al. 2021; Stange et al. 2021). Integrating genetic information in biodiversity assessments can lead to a different and more effective area selection for the protection of species (Hanson et al. 2020; Mastretta‐Yanes et al. 2024). Due to the idiosyncratic nature of evolutionary and population genetic histories, population genetic data cannot be easily inferred from simple parameters like geographic distance or by environmental data (Hanson et al. 2021, 2017). Taking genetic data into account could save costs and time by preemptively preserving gene flow and particularly valuable populations, prioritising intensive management, habitat restoration and efforts to re‐establish exchange with these populations.

One reason behind the neglect of genetic diversity has been the lack of awareness about its status as well as the lack of simple indicators and language for decision‐makers (Coates et al. 2018; Hoban et al. 2020; Laikre et al. 2010). During recent decades, however, knowledge on genetic diversity has grown substantially, and the application of genetic methods is becoming increasingly affordable. Therefore, commitments for the conservation of genetic diversity have become more ambitious (Hoban et al. 2023), and scientists are now exploring ways to incorporate genetic diversity into conservation strategies (Hoban et al. 2025).

Key Biodiversity Areas (KBAs) are a globally recognised standard to identify sites that are particularly valuable for conservation of biodiversity and contribute significantly to the persistence of biodiversity (Edgar et al. 2008; Eken et al. 2004; IUCN 2016). The coverage of KBAs by protected areas is an official indicator of the sustainable development goals (SDG) and, therefore, of high importance in the process of protected area designation (Nania et al. 2024). To qualify as a KBA, a proponent must provide evidence that the proposed area meets the thresholds of the IUCN KBA criteria (IUCN 2016). These criteria include, inter alia, the proportion of the global population of a species, with lower thresholds for threatened and higher thresholds for non‐threatened species. To measure this proportion of the global population size, the KBA standard allows the use of several metrics, including ‘distinct genetic diversity’. However, the latter metric has not yet been applied in practice due to a lack of sufficient testing and guidance. The IUCN KBA standard (IUCN 2016) defines distinct genetic diversity as ‘the proportion of a species' genetic diversity that is encompassed by a particular site’ and the subsequent guidance (IUCN 2020) highlights that it ‘differs from other assessment parameters in that it refers to the unique nature of a species' genetic diversity’. A site qualifies as a KBA if it exceeds a threshold proportion of a species' global genetic diversity, even if it would not trigger a KBA based on species population size data alone. To quantify distinct genetic diversity, the IUCN KBA standard proposes Analyses of Molecular Variance (AMOVA) or similar techniques that capture ‘distinct genetic diversity’ (IUCN 2016, 2020). The wording of the KBA standard ‘distinct genetic diversity’, however, incorporates two different concepts, ‘distinctiveness’ and ‘diversity’, both of which are important for biodiversity conservation. While genetic diversity within a population may be measured, for example, by the number of alleles or expected heterozygosity, genetic distinctiveness is measuring differentiation compared to other populations. These two concepts may be conflicting objectives and there are no valid arguments on how to prioritise between both concepts (Arponen 2012; Palmer and Fischer 2022). Instead of prioritising one of these aspects of genetic diversity, the recommendation is to assess both for a holistic understanding (Karanth et al. 2019). So far, the suitability of AMOVA or other methods to confirm that a site meets the KBA criteria has not been tested, nor has it been investigated which methods are most suitable to capture both genetic distinctiveness and diversity simultaneously.

We therefore tested the applicability of six methods for the identification of KBAs, three of which represent typical methods used in population genetics: AMOVA, *D*_est_ and effective population size (*N*_e_), and three biodiversity indices which are commonly applied to ecological communities: allelic overlap, Simpson's λ and a genetic derivation of average taxonomic distinctness (AvTD, Δ^+^) (Supp_Tab1). As the aim of KBA identification is to identify areas which contain a certain proportion of the global population (or genetic setup), we focused on allele frequencies rather than measures based on heterozygosity (F_ST_ and its derivations) as these are usually sensitive to allele frequency distribution and deviations from Hardy Weinberg Equilibrium (Jakobsson et al. 2013). While AMOVA and *D*_est_ are measures of genetic distinctiveness (Excoffier et al. 1992; Jost 2008), *N*_e_ and Simpson's λ can be considered measures of genetic diversity. Δ^+^ is used in community ecology to measure taxonomic distinctness. It simultaneously captures diversity and distinctness (Clarke and Warwick 1998; Schweiger et al. 2008) and has so far not been applied to genetic data although it should be applicable to allele frequencies in a similar way to species' frequencies in communities. Similarly, allelic overlap is derived from an ecological measure of beta diversity or ecological niche overlap (Czekanowski or Pianka index) and has not yet been used for genetic data. Areas exhibiting the lowest allelic overlap (i.e., sharing the fewest alleles with other areas) should be the most distinct. The reason for including ecological methods in our analyses is that we focused on allele frequency data which are the basis of any population genetic analyses (Hardy 1908) and thus akin to species abundance data. Clustering methods, which are commonly used in population genetics, were not considered because they do not allow quantification of distinct genetic diversity. However, they help to illustrate the performance of the different methods.

The aim of our study was to facilitate the use of distinct genetic diversity for the identification of KBAs by testing methods and providing guidance for its application. For this, we tested the performance of the methods based upon 30 published datasets, 15 using SNPs and 15 using microsatellites.

## 2 Material and Methods

As both SNP and microsatellite datasets are commonly used to analyse intraspecific genetic variance, we tested the performance of our six chosen analytical approaches on 30 published diploid datasets, of which 15 used SNPs (with an average of 184 SNP loci) and 15 microsatellite datasets (with an average of 31 microsatellite loci) (Supp_Tab2). Even though mtDNA datasets are still commonly used in phylogenetic studies, we did not include these in our analyses as they represent the maternal lineage only, are less variable, and are sensitive to hybridization. Each dataset was analysed with six methods: AMOVA (Excoffier et al. 1992), allelic overlap (Pianka 1973; Czekanowski 1913), Δ^+^ (Clarke and Warwick 1998), *D*_est_ (Jost 2008), λ corrected for sample size (Simpson 1949) and *N*_e_ (Wright 1931) (Supplement, Extended Methods). To apply all six methods, an R project was created that makes use of many packages that facilitate displaying results and working with genetic data and tables (Jombart 2008; Jombart and Ahmed 2011; R Core Team 2021; RStudio Team 2020; Wei and Simko 2021; Wickham et al. 2019; Wickham and Bryan 2023).

A key feature of KBAs is that they need to be manageable units (IUCN 2016). In practice, KBAs are therefore unlikely to be delineated based upon the genetic structure of a single species. Instead, proposed KBAs will align with existing management units, such as protected areas, Important Bird Areas, topographic features or political boundaries (IUCN 2016). Therefore, we did not perform analyses based on detected genetic clusters because proposed areas do not correspond to the biological structure of the species. The datasets we used were originally not intended to identify KBAs and therefore do not meet the requirements for a proper KBA identification process (IUCN 2020) as they do not cover the whole range of a species and do not specify areas that may meet the KBA thresholds. As our aim was to test the performance of different metrics and not to identify real‐world KBAs, we treated the localities provided by the datasets as proxies for KBAs. For this reason, we refer to ‘areas’ rather than ‘potential KBAs’. Nevertheless, the datasets include enough individuals from different localities making it possible to analyse genetic variation between areas and test the performance of different methods to measure distinct genetic diversity.

For better comparability between the six methods, all datasets were prepared in the same way. Sites with fewer than 30 individuals were removed from the analysis as this can greatly reduce negative and unrealistically low *N*_e_ values (England et al. 2006; Gargiulo et al. 2023). Individuals with > 20% missing data were removed from the dataset to prevent overestimated *N*_e_ (Gargiulo et al. 2023). For loci with missing genotypes, the missing allele counts were replaced with the mean of the observed alleles at that locus across all individuals in the dataset.

To explore similarities between the different approaches, correlations between the results of all six methods, allelic overlap, AMOVA, Δ^+^, *D*_est_, *N*_e_ and λ_cor_ were calculated in R. Correlations with allelic richness were additionally calculated (Goudet and Jombart 2022). Two outliers were removed from AMOVA results, as they lay far beyond the 1.5 × the interquartile range (IQR) of the distribution. Including these points did not substantially change the results. A Kendall correlation was chosen as it is more robust and slightly more efficient than Spearman's rank correlation and does not assume a normal data distribution like the Pearson correlation (Croux and Dehon 2010; Field et al. 2012). Correlations between allelic overlap, allelic richness, AMOVA, Δ^+^, *D*_est_, *N*_e_ and λ_cor_ are based on different sample sizes, since *N*_e_ could not be calculated for some areas.

We tested all results against two KBA criteria, A1b (> 1% of the global distinct genetic diversity occurs at this site) and B1 (> 10% of the global distinct genetic diversity occurs at this site). For each of the six methods, the proportion of distinct genetic diversity was calculated as the simple proportion of distinct genetic diversity at each location of the sum of all locations, as proposed by the KBA standard for AMOVA results (IUCN 2020). Areas lacking *N*_e_ were allocated the median of remaining areas to enable the application of KBA criteria without inflating the proportion of the remaining sites.

### 2.1 Case Studies

To illustrate the results and coverage of different genetic clusters, Structure analyses were conducted in addition to the calculation of the five metrics for two case studies: the Chinook salmon ( *Oncorhynchus tshawytscha* ; Gomez‐Uchida et al. 2019) as well as a hitherto unpublished dataset of the Tenerife Short‐winged Bush‐cricket (*Ariagona margaritae*) (Gronefeld et al. 2023). The site selection was based upon KBA criterion B1. The results were processed in R (R Core Team 2021) using several packages to obtain more information for the evaluation of the selected KBAs (Jombart 2008; Jombart and Ahmed 2011; Wickham et al. 2019). The maps were created using arcgis pro (ESRI 2024).

For the Chinook salmon ( *Oncorhynchus tshawytscha* ) Structure 2.3.4 was executed with 20 iterations for each K value and 20,000 Markov Chain Monte Carlo repeats after a burn‐in period of 10,000, followed by the application of Clumpak (Kopelman et al. 2015; Pritchard et al. 2000). We used these short Structure runs as Structure analyses were already included in the original paper (Gomez‐Uchida et al. 2018) and we only controlled for congruence with their results. The model was left at the default (Porras‐Hurtado et al. 2013).

Specimens of the geographically restricted Tenerife Short‐winged Bush‐cricket (*Ariagona margaritae* Kraus, 1892, Tettigoniidae, Orthoptera) were collected 2010–2023 on Tenerife and El Hierro. DNA was extracted using the Qiagen DNeasy Blood & Tissue kit. ddRADseq libraries were prepared for paired‐end sequencing on a High‐Output Flow cell of an Illumina NextSeq platform (2 × 75 bp) (Suchan et al. 2023). Stacks 2.6.6 was used to demultiplex, filter and trim raw reads to 65 bp and to create an assembly and a catalogue of loci to finally identify SNPs (*n* = 64, −*p* 150, −*r* 1) (Catchen et al. 2013). Default settings were maintained as recommended (Paris et al. 2017). Individuals containing more than 20% of missing data were removed from the analysis. The resulting dataset comprised 108 individuals and 5198 loci. Structure 2.3.4 was executed with 20 iterations for each *K* value and 1,000,000 Markov Chain Monte Carlo repeats after a burn‐in period of 100,000. No area was excluded from the analysis as each had ≥ 20 sequenced individuals. The allelic overlap method was omitted for this dataset due to extensive calculation times. For *N*_e_, the smallest possible natural number was added to transform all *N*_e_ into positive numbers. Apart from that, this dataset was analysed in the same way as the previously used datasets.

## 3 Results

**FIGURE 1:**
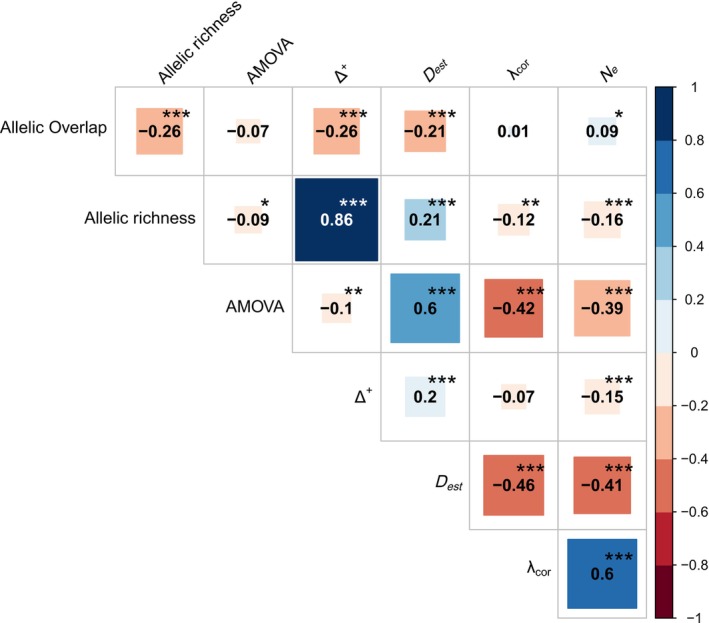
Correlations between Allelic Overlap, Allelic richness, AMOVA, Δ^+^, *D*_est_, λ_cor_ and *N*_e_. Each box representing the correlation between two methods comprises correlation coefficient and *p*‐value. ***p < 0.001, **p < 0.01, *p < 0.05.

The application of KBA criteria resulted, irrespective of the method used, in approximately the same ratio of sites being identified under criterion B1 and under criterion A1b (Supp_Fig1). The methods measuring distinctiveness (AMOVA and *D*_est_) correlated negatively with methods measuring genetic diversity (λ_cor_ and *N*_e_) but positively with each other (Figure 1). The positive correlation between the two methods measuring diversity was strong, but λ_cor_ reached a plateau at a value of 1 rapidly (Supp_Fig2), so that it did not capture variation between sites sufficiently for prioritisation. *N*_e_ sometimes resulted in negative or infinite values, making the calculation of ratios complicated. Its right‐skewed and heavy‐tailed distribution (Supp_Fig3) resulted in a few sites having comparatively high values and a lower number of identified KBAs (Supp_Fig1). Δ^+^ showed a very strong positive correlation with allelic richness. Apart from that, Δ^+^ and allelic overlap did not correlate with methods that cover only genetic diversity or genetic distinctiveness but correlated negatively with each other (Figure 1). Among the six metrics tested in this study, allelic overlap had by far the longest computation times.

**FIGURE 2:**
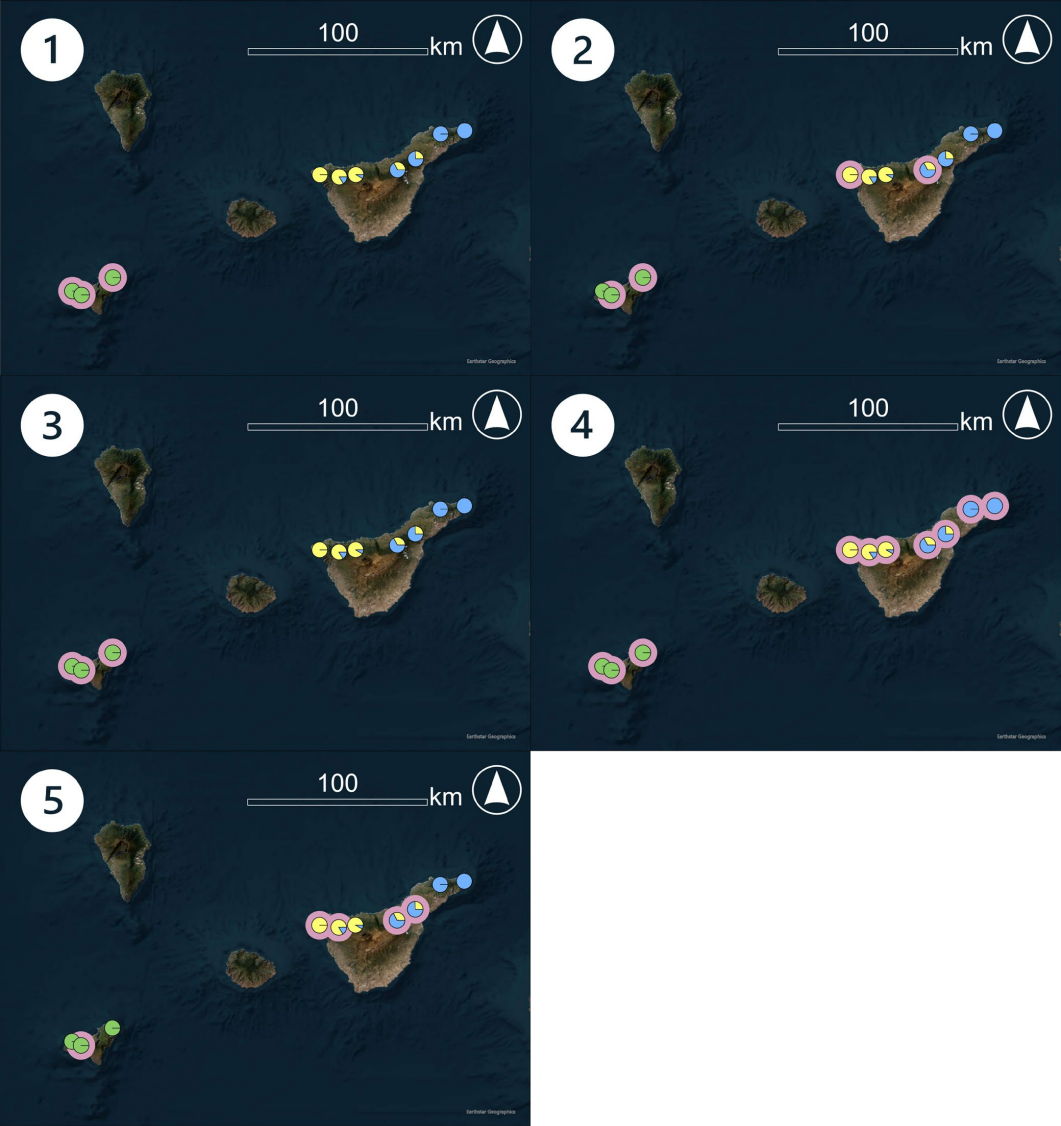
Genetic structure based upon structure and identified KBAs of the Tenerife Short‐winged Bush‐cricket (*Ariagona margaritae* Kraus, 1892) on the Canary Islands, Spain. (1–5) Pie charts represent the proportional structure cluster assignment for each area, with each colour indicating a distinct cluster, and pink frames outlining areas identified as KBAs by each method, (1) AMOVA, (2) Δ^+^, (3) *D*_est_, (4) λ_cor_ and (5) *N*_e_.

**FIGURE 3:**
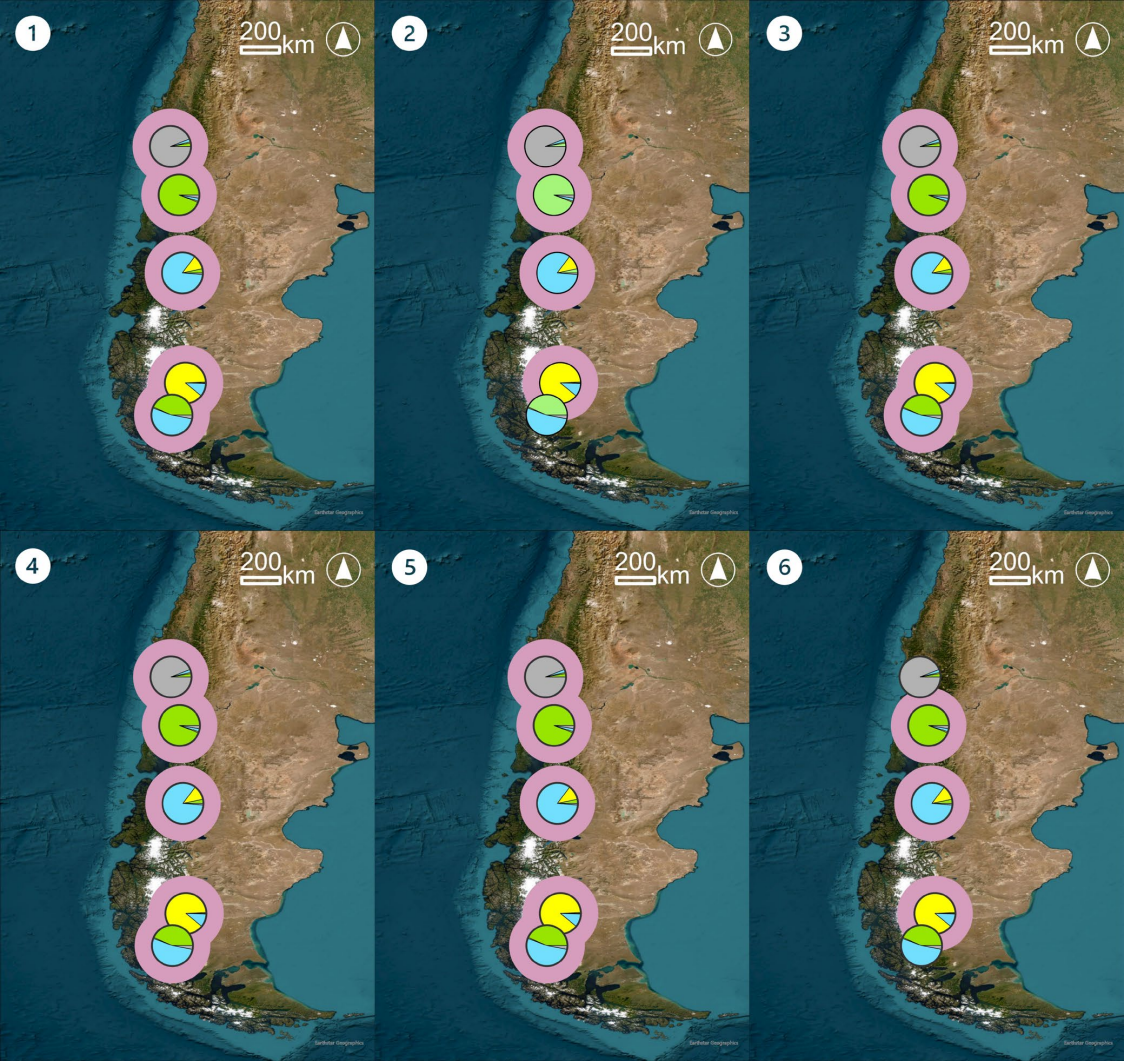
Genetic structure based upon structure and identified KBAs of the Chinook salmon ( *Oncorhynchus tshawytscha* Walbaum, 1792) in Chile and Argentina. (1–5) Pie charts represent the proportional structure cluster assignment for each area, with each colour indicating a distinct cluster, and pink frames outlining areas identified as KBAs by each method, (1) Allelic Overlap, (2) AMOVA, (3) Δ^+^, (4) *D*_est_, (5) λ_cor_ and (6) *N*_e_.

The performance of the methods is best illustrated by the two case studies (Figures 2 and 3). In the Tenerife Short‐winged Bush‐cricket (*Ariagona margaritae*), AMOVA and *D*_est_ selected only the most genetically distinct areas on the island El Hierro as KBAs; λ_cor_ selected all areas while Δ^+^ and *N*_e_ selected approximately half of all areas covering all three Structure clusters. In the Chinook salmon ( *Oncorhynchus tshawytscha* ), nearly all methods selected all structure clusters, except for *N*_e_, which missed genetically unique clusters.

## 4 Discussion

The KBA identification process aims to protect the ‘unique nature of a species' genetic diversity’ (IUCN 2020) with the goal to reduce the extinction risk of species. It is crucial to ensure that both genetically distinct populations and genetic diversity of the species are adequately covered. While protecting genetic diversity ensures adaptability, increases mean fitness and decreases inbreeding within populations (Frankham et al. 2006), the protection of genetic distinctiveness ensures the evolutionary potential and trajectory across its geographic distribution (Forester et al. 2022). A distinct allele composition may contain unique adaptations to local environments, which should be of conservation concern (Ceballos et al. 2020). These distinct alleles might be more prone to vanish (Maruyama and Fuerst 1985), while possibly contributing more to the overall survival of the species than many widespread alleles. If KBAs fail to protect genetic diversity, a species might lose its adaptive potential, leading to an increased extinction risk (Weeks et al. 2016). Even genetically depauperate populations, for example, caused by genetic bottlenecks, might be genetically distinct due to unique allele frequencies (Schulte et al. 2013) but might not be genetically diverse enough to persist for a long time. Protecting a wide range of genetically unique areas harbouring a maximum of within‐area genetic diversity could successfully minimise the overall risk of losing genetic diversity, akin to a diversified portfolio entailing fewer financial risks (Figge 2004). Therefore, any method for identifying KBAs based upon genetic data must ideally consider both aspects, genetic distinctiveness and genetic diversity. In addition, the method also needs to be evaluated based upon its applicability and how well the results can be interpreted (Table 1).

**TABLE 1:** Comparison between the metrics allelic overlap, AMOVA, Δ^+^, *D*_est_, λ_cor_ and *N*_e_.

	Allelic overlap	AMOVA	Δ^+^	*D*_est_	λ_cor_	*N*_e_
Captures	Genetic distinctiveness	Genetic distinctiveness	Genetic diversity and distinctiveness	Genetic distinctiveness	Genetic diversity	Genetic diversity
Is difficult to apply?	Yes (long calculation times)	No	No	No	No	Yes (min. 30 samples per area and few missing data)
Is difficult to interpret?	Yes (genetic distinctiveness is only captured indirectly)	Yes (is dependent on sample size)	No	No	Yes (almost never recognises differences between areas)	Yes (many outliers)
Could be used to identify KBAs?	No	No	Yes, recommended	Yes, not recommended (does not consider diversity)	No	No

### 4.1 Applicability

The method proposed by the KBA standard, AMOVA, can be calculated quickly and easily by many different established programmes (Excoffier and Lischer 2010; Peakall and Smouse 2012; Kamvar et al. 2014). However, adopting the method to identify KBAs is problematic because distinct genetic diversity estimates are required for each area. AMOVA provides only overall variation among all areas combined and separate pairwise analyses are needed for each area. This approach introduces a methodological problem: the Sum of Squares depends on sample size, whereas the Mean of Squares cannot be compared across separate analyses due to the division with a different number of degrees of freedom (Bird et al. 2011). Allelic overlap is computationally too intensive and should not be used as a measure of distinctiveness. Rare and private alleles have a much weaker influence on allelic overlap than abundant alleles, but they can be important to estimate genetic distinctiveness. Therefore, to measure and compare genetic distinctiveness between potential KBAs it is recommended to use *D*_est_ instead of AMOVA or allelic overlap. The calculation of *D*_est_ is easier and faster than AMOVA and is not biassed by sample size.

Both methods measuring diversity (λ_cor_ and *N*_e_) cannot be deemed as suitable methods for KBA identification. The calculation of λ_cor_ is fast and easy, but areas with only slightly higher genetic diversity than an *N*_e_ > 20 can hardly be distinguished by λ_cor_, a distinction which is relevant in practice. To avoid this problem, other methods to correct λ or another index for genetic diversity should be used.

The application of *N*_e_ is complicated, as there is a variety in methods, programmes and settings within the programmes. Methods which are good in precision have many data requirements. Apart from the LD method we used, the temporal method is a good choice to create reliable results, but the populations should be isolated, not migrating; there should be one to three generations between time samples, in addition to a recommended minimum of 30 individuals per time sample (Gilbert and Whitlock 2015; Olah et al. 2021). The LD method can be applied more easily to more datasets, but achieving the recommended 30 samples per site for a good quality of *N*_e_ estimates can be challenging in practice (England et al. 2006; Gargiulo et al. 2023), particularly when dealing with rare or threatened species.

While sequencing the genome of a single individual can provide estimates of the population's *N*_e_ (Doña and Johnson 2022), which is particularly valuable for rare or threatened species (Vitorino et al. 2019), this method estimates historical *N*_e_ and does not reliably reflect contemporary *N*_e_, which is more relevant for conservation management and necessary for KBA identification. It has also been proposed to estimate *N*_e_ from census population sizes (*N*_c_) using typical *N*_e_/*N*_c_ ratios to guide conservation decisions (Hoban et al. 2025; O'Brien et al. 2022). For KBA identification such proxies are not useful because numerous alternative metrics have already been defined in the KBA standard (IUCN 2016). If population size estimates exist, these can readily be used without inferring effective population sizes.

Akin to previous studies, our analysis indicates that *N*_e_ estimate distributions are right skewed with only a few very high values and many low ones (Mastretta‐Yanes et al. 2024). When calculating proportions, sites with small *N*_e_ could be excluded even though they are valuable, as can be seen in the example of the Chinook salmon ( *Oncorhynchus tshawytscha* ). Furthermore, negative or infinite *N*_e_ estimates occurred regularly and made the application of KBA criteria difficult. Even though *N*_e_ is valuable in monitoring the viability of populations and for conservation prioritisation, its tendency to produce outliers makes it extremely hard to interpret in the KBA context that depends on ratios.

Δ^+^ is applied easily as it is calculated quickly; the code to calculate it is not long or complicated. Δ^+^ has the advantage of being independent of sample size (Clarke and Warwick 1998, 2001). It also does not require Hardy–Weinberg‐Equilibrium as it is based on allele frequencies and is easy to interpret. Overall, *D*_est_ and Δ^+^ are the only measures that can be applied and interpreted reliably.

### 4.2 Performance in Identifying Distinct Genetic Diversity

Our results confirm that diversity and distinctiveness can be conflicting objectives (Figure 1) and that AMOVA, *D*_est_, and allelic overlap fail to capture genetic diversity, which could negatively affect the long‐term adaptability and fitness of the species. The shortcomings of using a method measuring distinctiveness only are illustrated by the example of the Tenerife Short‐winged Bush‐cricket (*Ariagona margaritae*). The KBAs identified using AMOVA and *D*_est_ would maintain only a single structure cluster. This is even more significant as it has been proposed that the populations on the different islands may represent different species (H. López pers. comm.). Such uncertainties regarding the taxonomic status of genetic lineages are not unusual and missing out on some potential future species or evolutionary lineages would not be in line with global biodiversity targets.

Δ^+^ preferentially selects KBAs with high allelic richness and low allelic overlap (Figure 1). Δ^+^ may, therefore, resolve the conflict between protecting genetic diversity and genetic distinctiveness. It allows the preservation of many different alleles, which would not be possible with a method that considers distinctiveness alone. Additionally, it covers rare alleles, which would be neglected with the diversity measuring methods *N*_e_ and λ. Consequently, the objective of the KBA standard to reduce extinction risk is more effectively met using Δ^+^. Nevertheless, the effectiveness of identified KBAs using Δ^+^ should be investigated further with complete datasets from real‐world examples. Potentially, Δ^+^ may also leave important clusters unprotected. Testing each area independently may fail to reach an optimal coverage of a species' genetic diversity as Δ^+^ might select several areas with high distinct genetic diversity even if these are genetically similar. A solution to overcome this shortfall could be testing different combinations of several areas to obtain a more holistic approach to preserve genetic diversity.

### 4.3 From Theory to Practice

Any estimate of distinct genetic diversity across areas requires sufficient sampling across the species' range to avoid misinterpretation, which remains challenging for many taxa. However, the costs and effort of obtaining a genetic data set still represent key limitations for application in practice. The challenges are particularly pronounced in the Global South, including many megadiverse countries, where limited funding, infrastructure and access to genomic technologies constrain the implementation of large‐scale genetic studies (Vilaça et al. 2024). A more representative picture of genetic diversity could nonetheless be achieved through stratified random sampling across environmental gradients, such as elevation in mountain regions. Indeed, even the data sets used in this study would not be suitable for KBA identification even though they provided a useful basis for the evaluation of the methods. In addition to the importance of the calculation method, the threshold value has a major influence on how many areas are selected. As the threshold values differ among Red List categories, it is critical that the Red List assessment is available and up to date.

Using distinct genetic diversity as an additional layer of decision making in KBA identification is important, because genetic diversity and species diversity do not always correlate positively (Kahilainen et al. 2014) and may even have an inverse relationship in some cases (Schmidt et al. 2022). Preserving areas with high distinct genetic diversity is thus crucial to ensure the conservation of the full evolutionary potential of a species. Moreover, a recent study has shown that land managers may generally be supportive of integrating genetic information into conservation management (Minter et al. 2021). We hope through improving KBA identification with the distinct genetic diversity metric, we can support scientists, policy makers and land managers to integrate genetic diversity into conservation planning, ensuring the long‐term evolutionary resilience of species.

## 5 Conclusions

Δ^+^ is a promising method for identifying KBAs because it captures a high allelic richness and distinct alleles, and it is quick and easy to apply and interpret. Further testing is needed to validate its usefulness for a comprehensive dataset covering the complete range of a species. A more holistic approach that considers the contribution of a set of KBAs to total distinct genetic diversity should be compared with the standard approach, which treats each KBA independently. *D*_est_ is also applied fast and easily interpreted, but it would not identify KBAs maximising the number of different alleles. All other methods: allelic overlap, AMOVA, λ_cor_ and *N*_e_ are either difficult to apply, to interpret or both and are therefore not suitable for KBA identification.

## Author Contributions

Sarah Christin Gronefeld designed methodology, analysed data and wrote the manuscript. Heriberto López organised the permits to collect specimens, supervised field work, collected specimens and translated our abstract. Robin Schmidt supported the lab work, gave advice on programmes and helped to analyse and evaluate the Tenerife Short‐winged Bush‐cricket (*Ariagona margaritae* Kraus, 1892) dataset. Axel Hochkirch initiated the project, conceived ideas and contributed to the draft. All authors gave final approval for publication.

## Funding

This project was funded by BMBF via Biodiversa+ (Grant number: 16LW0320).

## Disclosure

Benefit‐Sharing Statement: A research collaboration was developed with scientists from the country providing samples, who were included as a coauthor or mentioned in the Acknowledgments. As required by the collection permit we shared information on the collection points with the Cabildo Insular de El Hierro and the Cabildo Insular de Tenerife. We additionally provided the abstract in Spanish, which is the local language. The goal of this research is to facilitate conservation of distinct genetic diversity globally, which also aids the conservation of *Ariagona margaritae* on the Canary Islands. We are committed to international partnerships and sharing the benefits of our research.

## Ethics Statement

The authors have nothing to report.

## Consent

The authors have nothing to report.

## Conflicts of Interest

The authors declare no conflicts of interest.

## Supporting information


**Data S1:** men70094‐sup‐0001‐DataS1.pdf.

## Data Availability

Extended Methods, supplemental figures and tables: https://doi.org/10.5061/dryad.573n5tbhk; Code: https://github.com/TheC0der856/genetic_distinct_diversity.
