# Supplementary material for: Identifying Key Biodiversity Areas Based on Distinct Genetic Diversity
Source: Mol Ecol Resour. 2026 Jan 10;26(2):e70094. doi: 10.1111/1755-0998.70094 (PMC12789961; doi:10.1111/1755-0998.70094)
Supplement: Supplementary file 1 — Data S1: men70094‐sup‐0001‐DataS1.pdf. [file MEN-26-e70094-s001.pdf]

Supplemental Information for:

Identifying Key Biodiversity Areas based on distinct genetic diversity

Sarah Christin Gronefeld, Heriberto López, Robin Schmidt & Axel Hochkirch

Table of Contents

Extended Methods 1

Allelic overlap 1

AMOVA 1

Δ^+ 1

D_{est} 2

N_e 2

λ_{cor} 2

Figures and Tables 3

SuppFig1: Sites meeting KBA criteria B1 and A1b for Allelic Overlap, AMOVA, Δ^+, D_{est}, λ_{cor}, and N_e 3

SuppFig2: Correlation between λ_{cor} and N_e < 100, illustrating the rapid plateau of λ_{cor} at a value of 1 4

SuppFig3: Right-skewed and heavy-tailed distribution of N_e 5

SuppTab1: Descriptions of Methods. 6

SuppTab2: Microsatellite and SNP data sets. 7

SuppTab3: Collection data. 9

SuppTab4: Barcodes. Library names were abbreviated as 1_R1/1_R2 and 2_R1/2_R2, corresponding to sequencing runs 231201_NB501850_A_L1_4_AZJH_1 and 231201_NB501850_A_L1_4_AZJH_2. 12

References 14

Extended Methods

Allelic overlap

The R version of EcoSim was used to compute allelic overlap, which is denoted as the 'observed index' in the program's output (Gotelli & Entsminger, 2015; Gotelli et al., 2015). Instead of species abundances, allele abundances were chosen as input for the program. The default settings were kept as the use of the RA3 algorithm is recommended and there is no significant difference between using the Pianka and Czekanowski metrics (Burns et al., 2010; Castro-Arellano et al., 2010). Each single proposed area was compared to the rest of areas in the dataset.

AMOVA

The R package poppr was used to calculate AMOVA and λ (Kamvar et al., 2014). The percentage of variance between areas (i.e. the effect size) was used for KBA identification, because we aimed to quantify and compare variation between areas. Similar to allelic overlap a single area was compared to the rest of areas in the dataset. Contrary to the default setting for AMOVA, individuals with too many missing data points were removed instead of loci to ensure comparability and avoid error messages for microsatellite datasets.

Δ^+

The expected Δ^+ was calculated using the R package vegan (Oksanen et al., 2022). Instead of calculating the taxonomic distance between species in a community (Clarke & Warwick, 2001; Oksanen et al., 2022), the genetic distance between individuals in an area was calculated (equation 2). The genetic distance was calculated using the bitewise.dist() function of the poppr package, because it effectively calculates the distance between individuals in huge datasets and is the default distance method to calculate genetic distances in the AMOVA calculation of the poppr package (Kamvar et al., 2014).

$$\Delta^{+} = \frac{2}{n(n-1)} \sum_{i<j} d_{ij}$$

1

Δ^{+} = mean genetic distance between all individuals in one area

n = number of individuals in the area

d_{ij} = genetic distance between individual j and individual i

D_{est}

D_{est} was calculated using the HIERFSTAT package (Goudet & Jombart, 2022).

N_e

Contemporary N_e was calculated for each area using LD as recommended by most authors (Gilbert & Whitlock, 2015; Olah et al., 2020). The R interface of NEESTIMATOR, RDLNE, was used (Do et al., 2014; Robinson, 2019). A threshold of an allele frequency of 0.05 was selected to remove rare alleles. Although keeping rare alleles in the analyses is not guaranteed to skew the overall picture, they are known to cause a bias in N_e estimates (Marandel et al., 2020; Zachos et al., 2016). Negative and infinite N_e estimates were considered as missing information.

λ_{cor}

λ was calculated with POPPR (Kamvar et al., 2014). It was corrected for sample size as λ has a bias in estimating a higher genetic diversity for areas with a larger sample size (equation 1) (Grünwald et al., 2003; Grünwald et al., 2017).

$$\lambda_{\mathrm{cor}} = \frac{N}{N-1} * \lambda$$

2

N = number of samples

λ_{cor} = λ corrected for sample size

Figures and Tables

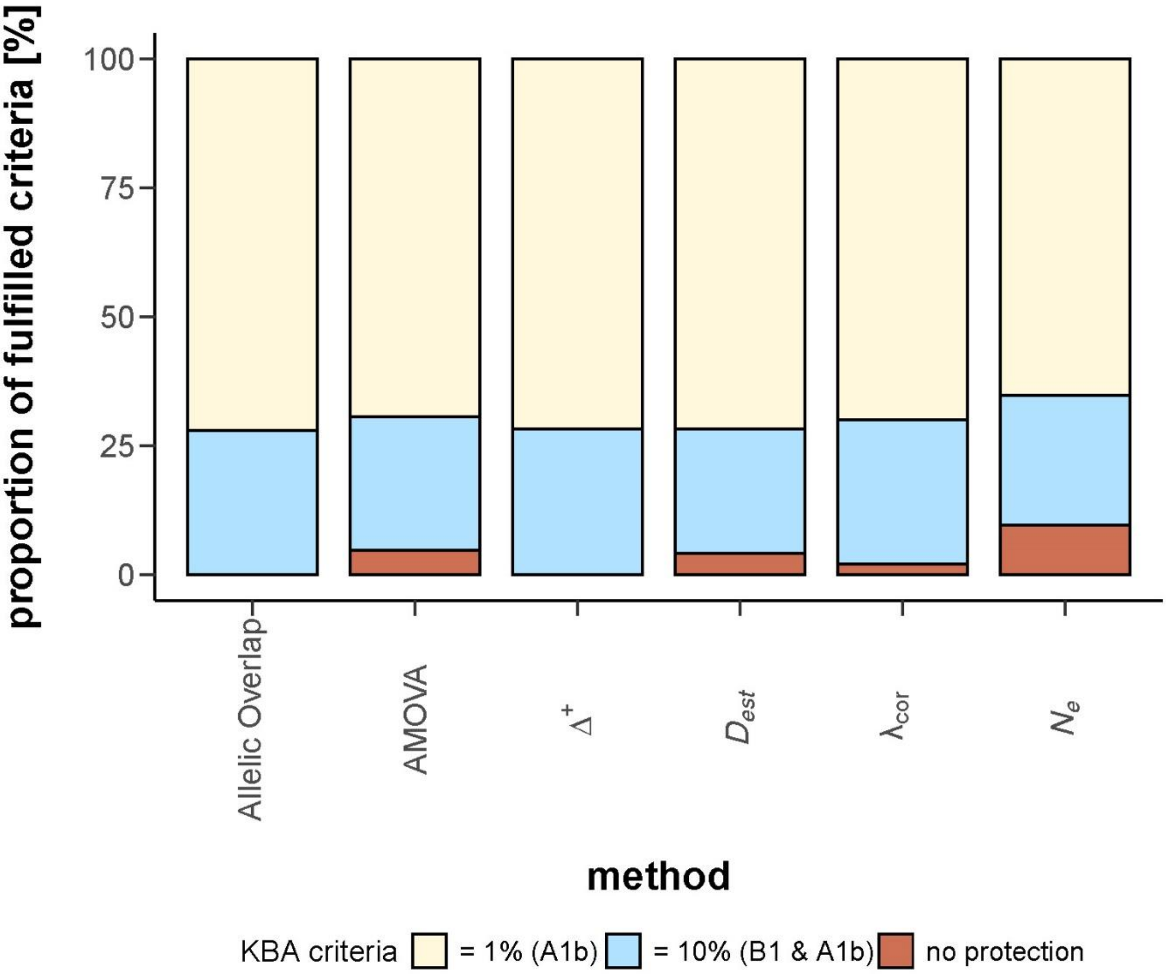

SuppFig1: Sites meeting KBA criteria B1 and A1b for Allelic Overlap, AMOVA, Δ^+, D_{est}, λ_{cor}, and N_e.

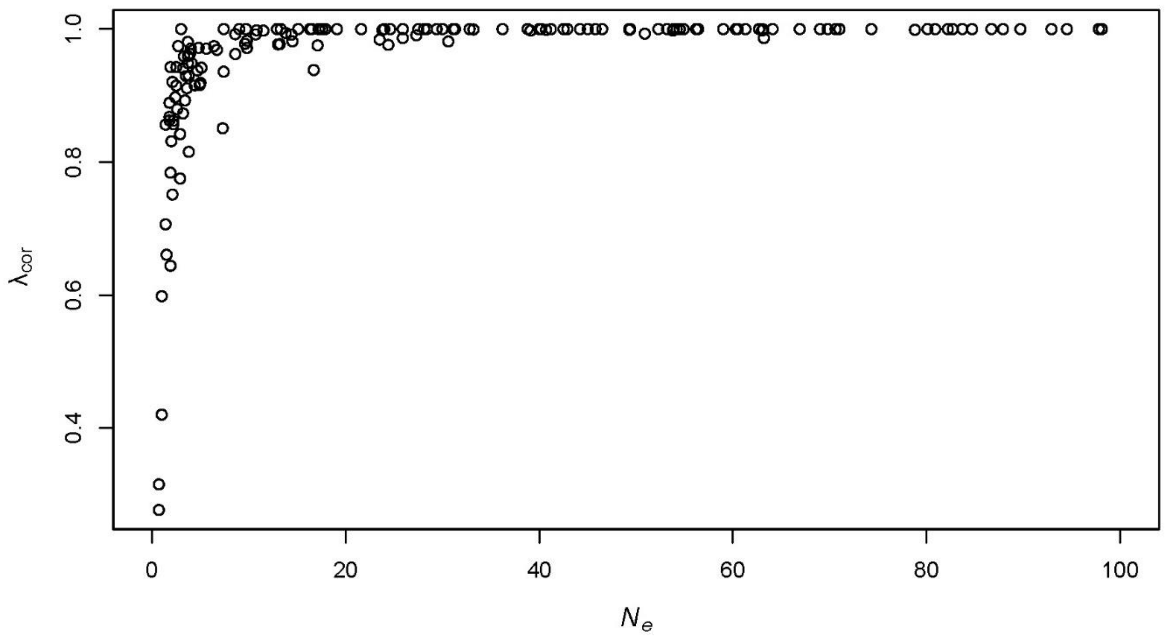

SuppFig2: Correlation between λ_{cor} and $N_e < 100$, illustrating the rapid plateau of λ_{cor} at a value of 1.

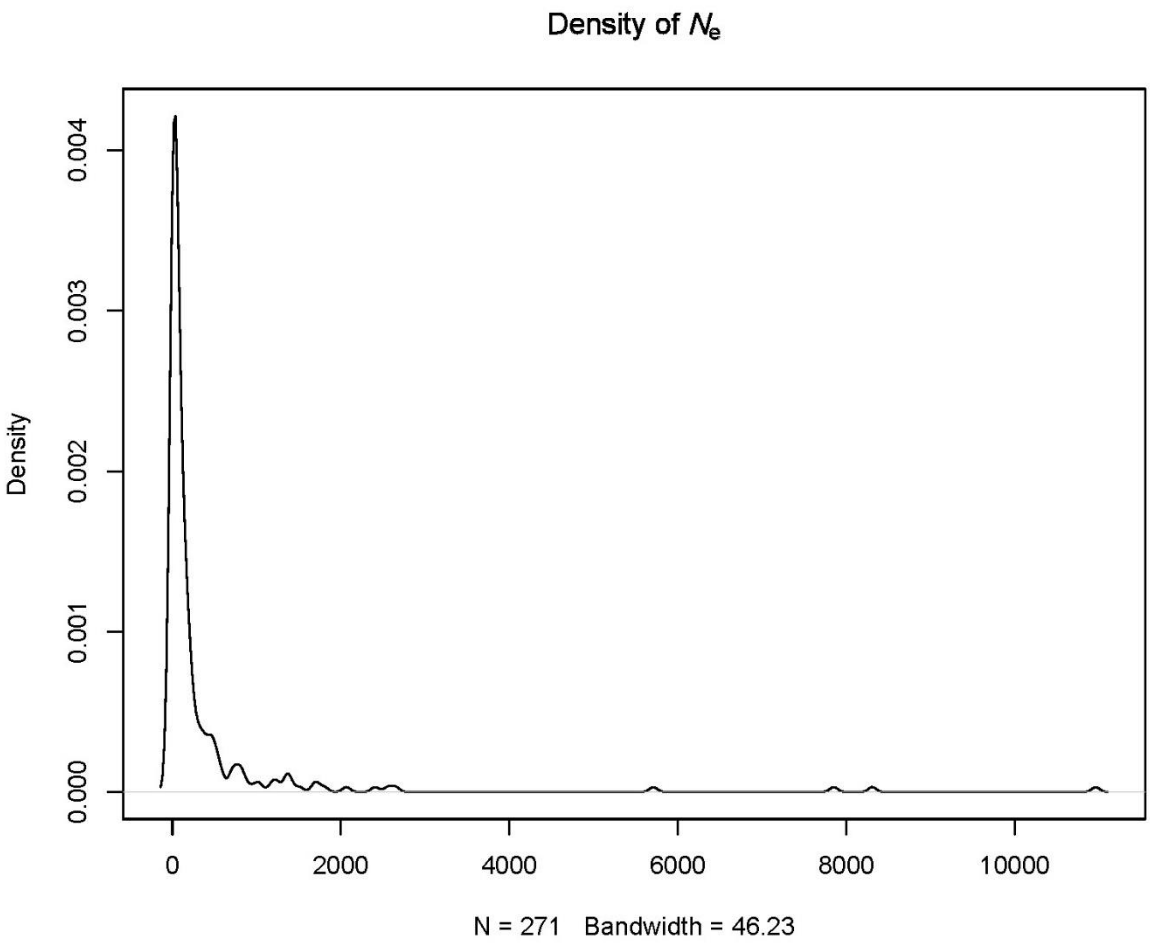

SuppFig3: Right-skewed and heavy-tailed distribution of N_e.

SuppTab1: Descriptions of Methods.

Method	Description
Allelic overlap	Allelic overlap is derived from an ecological measure of beta diversity or ecological niche overlap (Czekanowski or Pianka index). It calculates which areas have the most alleles in common with other areas.
AMOVA	AMOVA quantifies genetic differentiation among and within groups (Excoffier et al., 1992).
Δ^+	Δ^+ is used in community ecology to measure taxonomic distinctness. It simultaneously captures diversity and distinctness (Clarke & Warwick, 1998; Schweiger, 2008) due to its mathematical relationship with other indices. The index associated with Δ^+, taxonomic diversity (TD, Δ), can be expressed as a product of Simpson's λ and the taxonomic distance of each taxonomic level (Shimatani, 2001).
D_{est}	D_{est} evaluates the genetic differentiation among populations that is largely independent of within-population diversity. It is based directly on allele frequencies and does not rely on Hardy–Weinberg expectations (Jost, 2008).
Simpson's λ	Simpson's λ is an index that can be used to calculate genetic diversity and species diversity (Kamvar et al., 2014; Simpson, 1949).
N_e	N_e is currently included in the Kunming–Montreal Global Biodiversity Framework (GBF) monitoring framework as headline indicator A.4 (CBD, 2022), reflecting its importance for assessing genetic diversity and population viability. It has also been proposed as a valuable addition to assessing the extinction risk of a species in the context of the IUCN Red List (Garner et al., 2020; Hoban et al., 2021a; McLaughlin et al., 2025).

SuppTab2: Microsatellite and SNP data sets.

species	country	"areas" in the data set	"areas" analyzed	number of genetic markers	genetic marker	reference
Ambystoma bishopi	USA	14	5	9	micro-satellites	Wendt et al. 2021
Avicennia marina	Kenya	8	8	10	micro-satellites	Triest 2021
Cameraria ohridella	Austria, Belgium, Czech Republic, Denmark, England, France, Germany, Hungary, Italy, Lithuania, Netherlands, Poland, Romania, Spain, Switzerland, Bosnia, Bulgaria, Croatia, Slovenia, Albania, Greece, Macedonia	16	12	6	micro-satellites	Valade et al. 2010
Carcinus meanas	Canada, USA	10	7	11	micro-satellites	Lehnert et al. 2018
Cercidiphyllum japonicum	Japan	24	22	5	micro-satellites	Nakanishi 2022
Cymodocea nodosa	Portugal, Spain, Marocco	57	37	8	micro-satellites	Arnaud-Haond et al. 2014
Cystoseira amentaceae	Italy	12	8	8	micro-satellites	Buonomo R et al. 2016
Euphydryas aurina	Denmark	10	4	318	micro-satellites	Pertoldi 2022
Mytilus galloprovincialis	Croatia, Bosnia & Herzegovina, Montenegro, Albania	18	18	6	micro-satellites	Hamilton 2023
Pagophila eburnea	Denmark, Norway, Russia, Canada	5	3	15	micro-satellites	Charbonnel et al. 2022
Panthera leo	South Africa, Zimbabwe	24	5	28	micro-satellites	Miller et al. 2014
Posidonia oceanica	Spain, Italy, Cyprus, France, Tunisia, Malta, Croatia, Slovenia, Greece	39	38	7	micro-satellites	Arnaud-Haond et al. 2014
Pyura chilensis	Peru, Chile	26	19	8	micro-satellites	Quesada-Calderon et al. 2021
Syncerus caffer	Kamerun, Gabun, South Africa, Uganda, Kenia, Tanzania, Zimbabwe	17	8	20	micro-satellites	van Hooft et al. 2021
Varroa jacobsoni	Indonesia, Papua New Guinea	4	4	12	micro-satellites	Roberts, et al. 2015

Abies alba	Germany, Italy, France, Romania	8	8	137	SNP	Major et al. 2021
Argiope bruennichi	Algeria, Austria, Azerbaijan, Belgium, Bulgaria, China, Croatia, Czech Republic, Denmark, Estonia, Finland, France, Georgia, Germany, Greece, Hungary, Iran, Italy, Japan, Latvia, Lebanon, Lithuania, Macedonia, Morocco, Netherlands, Poland, Polynesia, Portugal, Romania, Russia, Slovakia, Spain, Sweden, Switzerland, Syria, Tunesia, Turkey, Ukraine, Uzbekistan	5	3	7	SNP	Krehenwinkel & Tautz 2013
Atriophallophorus winterbourni	New Zealand	6	3	34	SNP	Feijen 2022
Dracocephalum ruyschiana	Norway	10	3	92	SNP	Nygaard et al. 2022
Entosphenus tridentatus	Canada, USA	36	17	96	SNP	Hess et al. 2014
Frangula alnus	France, Belgium, Italy, Sweden	25	6	183	SNP	De Kort et al. 2015
Gadus morhua	Denmark, Canada, Iceland	29	19	935	SNP	Therkildsen et al. 2015
Melitaea cinxia	Finnland, Sweden, Estonia	4	4	42	SNP	Duplouy et al. 2018
Nilparvata lugens	China	5	5	89	SNP	Sun et al. 2015
Oncorhynchus mykiss	Chile	11	11	81	SNP	Benavente et al. 2016
Oncorhynchus tshawytscha	Chile, Argentina	9	5	172	SNP	Gomez-Uchida et al. 2019
Physeter macrocephalus	USA, Ecuador, Peru, Chile, Mexico, Russia, Canada, Colombia	11	4	36	SNP	Mesnick et al. 2010
Pinus halepensis	Spain, Italy, Morocco, Algeria, Tunisia, Greece, Israel	42	9	294	SNP	Ruiz Daniels et al. 2018
Salmo trutta	Sweden	27	26	96	SNP	Andersson et al. 2022
Tectona grandis	Indonesia	45	23	459	SNP	Prasetyo et al. 2020

SuppTab3: Collection data.

ID	collector	Longitude (WGS84)	Latitude (WGS84)	island	collection date
SG002	Sarah Gronefeld	-16.7851	28.32715	Tenerife	20 July 2023
SG003	Sarah Gronefeld	-16.7851	28.32715	Tenerife	20 July 2023
SG004	Sarah Gronefeld	-16.7851	28.32715	Tenerife	20 July 2023
SG005	Sarah Gronefeld	-16.7851	28.32715	Tenerife	20 July 2023
SG006	Sarah Gronefeld	-16.2583	28.55354	Tenerife	4 August 2023
SG007	Sarah Gronefeld	-16.493	28.36219	Tenerife	31 July 2023
SG008	Sarah Gronefeld	-16.493	28.36219	Tenerife	31 July 2023
SG009	Sarah Gronefeld	-16.493	28.36219	Tenerife	31 July 2023
SG010	Sarah Gronefeld	-16.493	28.36219	Tenerife	31 July 2023
SG011	Sarah Gronefeld	-16.493	28.36219	Tenerife	31 July 2023
SG012	Sarah Gronefeld	-16.493	28.36219	Tenerife	31 July 2023
SG013	Sarah Gronefeld	-16.493	28.36219	Tenerife	31 July 2023
SG014	Sarah Gronefeld	-16.493	28.36219	Tenerife	31 July 2023
SG016	Sarah Gronefeld	-16.493	28.36219	Tenerife	31 July 2023
SG017	Sarah Gronefeld	-16.493	28.36219	Tenerife	31 July 2023
SG019	Sarah Gronefeld	-16.493	28.36219	Tenerife	31 July 2023
SG026	Sarah Gronefeld	-18.075	27.73629	El Hierro	23 July 2023
SG027	Sarah Gronefeld	-18.075	27.73629	El Hierro	23 July 2023
SG028	Sarah Gronefeld	-18.075	27.73629	El Hierro	23 July 2023
SG029	Sarah Gronefeld	-18.075	27.73629	El Hierro	23 July 2023
SG030	Sarah Gronefeld & Heriberto López Hernández	-16.157	28.55799	Tenerife	2 August 2023
SG031	Sarah Gronefeld & Heriberto López Hernández	-16.157	28.55799	Tenerife	2 August 2023
SG032	Sarah Gronefeld	-16.8812	28.33792	Tenerife	28 July 2023
SG033	Sarah Gronefeld	-16.8812	28.33792	Tenerife	28 July 2023
SG034	Sarah Gronefeld	-16.8812	28.33792	Tenerife	28 July 2023
SG035	Sarah Gronefeld	-16.8812	28.33792	Tenerife	28 July 2023
SG036	Sarah Gronefeld	-16.8812	28.33792	Tenerife	28 July 2023
SG037	Sarah Gronefeld	-16.8812	28.33792	Tenerife	28 July 2023
SG038	Sarah Gronefeld	-16.8812	28.33792	Tenerife	28 July 2023
SG039	Sarah Gronefeld	-16.8812	28.33792	Tenerife	28 July 2023
SG040	Sarah Gronefeld	-16.8812	28.33792	Tenerife	28 July 2023
SG041	Sarah Gronefeld	-16.8812	28.33792	Tenerife	28 July 2023
SG046	Sarah Gronefeld	-16.157	28.55799	Tenerife	3 August 2023
SG047	Sarah Gronefeld	-16.157	28.55799	Tenerife	3 August 2023
SG049	Sarah Gronefeld	-16.157	28.55799	Tenerife	3 August 2023
SG050	Sarah Gronefeld	-16.157	28.55799	Tenerife	3 August 2023
SG051	Sarah Gronefeld	-16.7018	28.33852	Tenerife	19 July 2023
SG052	Sarah Gronefeld	-16.7018	28.33852	Tenerife	19 July 2023
SG053	Sarah Gronefeld	-16.7018	28.33852	Tenerife	19 July 2023
SG054	Sarah Gronefeld	-16.7018	28.33852	Tenerife	19 July 2023
SG055	Sarah Gronefeld	-16.7018	28.33852	Tenerife	19 July 2023
SG056	Sarah Gronefeld	-16.7018	28.33852	Tenerife	19 July 2023

SG057	Sarah Gronefeld	-16.7018	28.33852	Tenerife	19 July 2023
SG058	Sarah Gronefeld	-16.7018	28.33852	Tenerife	19 July 2023
SG059	Sarah Gronefeld	-16.7018	28.33852	Tenerife	19 July 2023
SG060	Sarah Gronefeld	-16.7018	28.33852	Tenerife	19 July 2023
SG061	Sarah Gronefeld	-16.7018	28.33852	Tenerife	19 July 2023
SG063	Sarah Gronefeld	-16.7018	28.33852	Tenerife	19 July 2023
SG065	Sarah Gronefeld	-16.7018	28.33852	Tenerife	19 July 2023
SG075	Sarah Gronefeld	-18.075	27.73629	El Hierro	24 July 2023
SG076	Sarah Gronefeld	-18.075	27.73629	El Hierro	24 July 2023
SG079	Sarah Gronefeld	-18.075	27.73629	El Hierro	24 July 2023
SG089	Sarah Gronefeld	-16.493	28.36219	Tenerife	1 August 2023
SG098	Sarah Gronefeld	-16.493	28.36219	Tenerife	1 August 2023
SG117	Sarah Gronefeld	-18.075	27.73629	El Hierro	25 July 2023
SG130	Sarah Gronefeld	-16.7851	28.32715	Tenerife	19 July 2023
SG131	Sarah Gronefeld	-16.7851	28.32715	Tenerife	19 July 2023
SG132	Sarah Gronefeld	-16.7851	28.32715	Tenerife	19 July 2023
SG133	Sarah Gronefeld	-16.7851	28.32715	Tenerife	19 July 2023
SG134	Sarah Gronefeld	-16.7851	28.32715	Tenerife	19 July 2023
SG135	Sarah Gronefeld	-16.7851	28.32715	Tenerife	19 July 2023
SG137	Sarah Gronefeld	-16.7851	28.32715	Tenerife	19 July 2023
SG139	Sarah Gronefeld	-16.7851	28.32715	Tenerife	19 July 2023
SG140	Sarah Gronefeld	-16.7851	28.32715	Tenerife	19 July 2023
SG141	Sarah Gronefeld	-16.7851	28.32715	Tenerife	19 July 2023
SG143	Sarah Gronefeld	-16.7851	28.32715	Tenerife	19 July 2023
SG149	Sarah Gronefeld & Heriberto López Hernández	-16.4023	28.41722	Tenerife	18 July 2023
SG150	Sarah Gronefeld & Heriberto López Hernández	-16.4023	28.41722	Tenerife	18 July 2023
SG151	Sarah Gronefeld & Heriberto López Hernández	-16.4023	28.41722	Tenerife	18 July 2023
SG154	Sarah Gronefeld & Heriberto López Hernández	-16.4023	28.41722	Tenerife	18 July 2023
SG155	Sarah Gronefeld & Heriberto López Hernández	-16.4023	28.41722	Tenerife	18 July 2023
SG156	Sarah Gronefeld & Heriberto López Hernández	-16.4023	28.41722	Tenerife	18 July 2023
SG158	Sarah Gronefeld & Heriberto López Hernández	-16.4023	28.41722	Tenerife	18 July 2023
SG161	Sarah Gronefeld & Heriberto López Hernández	-16.4023	28.41722	Tenerife	18 July 2023
SG162	Sarah Gronefeld & Heriberto López Hernández	-16.4023	28.41722	Tenerife	18 July 2023
SG164	Sarah Gronefeld & Heriberto López Hernández	-16.4023	28.41722	Tenerife	18 July 2023
SG168	Sarah Gronefeld & Heriberto López Hernández	-16.4023	28.41722	Tenerife	18 July 2023
SG170	Sarah Gronefeld & Heriberto López Hernández	-16.4023	28.41722	Tenerife	18 July 2023
T018	Tobias Schulte-Middelmann	-16.3896	28.4561	Tenerife	14 July 2015

T019	Tobias Schulte-Middelmann	-16.3896	28.4561	Tenerife	14 July 2015
T020	Tobias Schulte-Middelmann	-16.3896	28.4561	Tenerife	14 July 2015
T021	Tobias Schulte-Middelmann	-16.3896	28.4561	Tenerife	14 July 2015
T029	Tobias Schulte-Middelmann	-16.4432	28.43516	Tenerife	30 July 2015
T041	Tobias Schulte-Middelmann	-16.2771	28.54167	Tenerife	22 July 2015
T043	Tobias Schulte-Middelmann	-16.2771	28.54167	Tenerife	22 July 2015
T048	Tobias Schulte-Middelmann	-16.4432	28.43516	Tenerife	16 July 2015
T049	Tobias Schulte-Middelmann	-16.4432	28.43516	Tenerife	16 July 2015
T050	Tobias Schulte-Middelmann	-16.4432	28.43516	Tenerife	16 July 2015
T061	Tobias Schulte-Middelmann	-16.2771	28.54167	Tenerife	22 July 2015
T081	Tobias Schulte-Middelmann	-16.2771	28.55774	Tenerife	22 July 2015
T082	Tobias Schulte-Middelmann	-16.2787	28.5521	Tenerife	22 July 2015
T111	Heriberto López Hernández	-18.1204	27.75601	El Hierro	15 June 2011
T112	Heriberto López Hernández	-18.1204	27.75601	El Hierro	15 June 2011
T113	Heriberto López Hernández	-18.1204	27.75601	El Hierro	16 July 2011
T114	Heriberto López Hernández	-18.1204	27.75601	El Hierro	16 July 2011
T116	Heriberto López Hernández	-18.1204	27.75601	El Hierro	16 July 2011
T118	Heriberto López Hernández	-18.1204	27.75601	El Hierro	16 July 2011
T119	Heriberto López Hernández	-18.1204	27.75601	El Hierro	16 July 2011
T130	Heriberto López Hernández & Elena Morales	-17.9182	27.82353	El Hierro	24 July 2010
T132	Heriberto López Hernández & Elena Morales	-17.9182	27.82353	El Hierro	24 July 2010
T139	Heriberto López Hernández & Elena Morales	-17.9182	27.82353	El Hierro	24 July 2010
T142	Heriberto López Hernández & Elena Morales	-17.9182	27.82353	El Hierro	24 July 2010
T143	Heriberto López Hernández & Elena Morales	-17.9182	27.82353	El Hierro	24 July 2010
T163	Heriberto López Hernández & Elena Morales	-17.9182	27.82353	El Hierro	24 July 2010
T164	Heriberto López Hernández & Elena Morales	-17.9182	27.82353	El Hierro	24 July 2010
T167	Heriberto López Hernández & Elena Morales	-18.075	27.73629	El Hierro	23 July 2010
T169	Heriberto López Hernández & Elena Morales	-18.075	27.73629	El Hierro	23 July 2010
T170	Heriberto López Hernández & Elena Morales	-18.075	27.73629	El Hierro	23 July 2010

SuppTab4: Barcodes. Library names were abbreviated as 1_R1/1_R2 and 2_R1/2_R2, corresponding to sequencing runs 231201_NB501850_A_L1_4_AZJH_1 and 231201_NB501850_A_L1_4_AZJH_2.

ID	Barcodes	Library (forward)	Library (reverse)
SG002	ACGG	1_R1	1_R2
SG003	CATCG	1_R1	1_R2
SG004	TGTGCA	1_R1	1_R2
SG005	GTACGT	1_R1	1_R2
SG006	GGTAGCA	1_R1	1_R2
SG007	AATTGCG	1_R1	1_R2
SG008	AGAATGCA	1_R1	1_R2
SG009	GGTCTT	1_R1	1_R2
SG010	TGCT	1_R1	1_R2
SG011	ATCGA	1_R1	1_R2
SG012	TTGACA	1_R1	1_R2
SG013	TAGGCT	1_R1	1_R2
SG014	GTGACCA	1_R1	1_R2
SG016	GAATAGCA	1_R1	1_R2
SG017	TGCT	2_R1	2_R2
SG019	CGAG	2_R1	2_R2
SG026	CAAGTAGA	1_R1	1_R2
SG027	CATA	1_R1	1_R2
SG028	TCGAA	1_R1	1_R2
SG029	AGCTGA	1_R1	1_R2
SG030	GGCTAG	1_R1	1_R2
SG031	TTATGCA	1_R1	1_R2
SG032	TCAGCAG	1_R1	1_R2
SG033	ATGAGACA	1_R1	1_R2
SG034	GCAAGAAT	1_R1	1_R2
SG035	CGAG	1_R1	1_R2
SG036	ACCTG	1_R1	1_R2
SG037	TGGCAA	1_R1	1_R2
SG038	CATGTA	1_R1	1_R2
SG039	ATTGGCA	1_R1	1_R2
SG040	CAGTGCA	1_R1	1_R2
SG041	TGCCACCA	1_R1	1_R2
SG046	ACCTACCG	1_R1	1_R2
SG047	GCTT	1_R1	1_R2
SG049	CTATCG	1_R1	1_R2
SG050	ATTCGG	1_R1	1_R2
SG051	TGGTACA	1_R1	1_R2
SG052	GTACCGA	1_R1	1_R2
SG053	ATAGAGCA	1_R1	1_R2
SG054	CTACCACG	1_R1	1_R2
SG055	ATCA	1_R1	1_R2
SG056	CGCTA	1_R1	1_R2
SG057	GCTGAA	1_R1	1_R2
SG058	TGACCT	1_R1	1_R2

SG059	GACCTCA	1_R1	1_R2
SG060	TGTAACG	1_R1	1_R2
SG061	CATCG	2_R1	2_R2
SG063	ATCGA	2_R1	2_R2
SG065	TCGAA	2_R1	2_R2
SG075	ACTCGCCA	1_R1	1_R2
SG076	TGTGCA	2_R1	2_R2
SG079	TGGCAA	2_R1	2_R2
SG089	GACTCT	2_R1	2_R2
SG098	CATCCG	2_R1	2_R2
SG117	GTACGT	2_R1	2_R2
SG130	TAGAACGA	1_R1	1_R2
SG131	GACG	1_R1	1_R2
SG132	CCTGA	1_R1	1_R2
SG133	TTCCGA	1_R1	1_R2
SG134	GCTACT	1_R1	1_R2
SG135	TGTGCCA	1_R1	1_R2
SG137	CGATGT	2_R1	2_R2
SG139	GGTAGCA	2_R1	2_R2
SG140	GTGACCA	2_R1	2_R2
SG141	TTATGCA	2_R1	2_R2
SG143	TGGTACA	2_R1	2_R2
SG149	TACGATA	1_R1	1_R2
SG150	TAGGAACA	1_R1	1_R2
SG151	AGCAGTAA	1_R1	1_R2
SG154	GACTCT	1_R1	1_R2
SG155	TCGGTA	1_R1	1_R2
SG156	TAGACCG	1_R1	1_R2
SG158	GATACGAA	1_R1	1_R2
SG161	AGACTCG	2_R1	2_R2
SG162	AATTGCG	2_R1	2_R2
SG164	TCAGCAG	2_R1	2_R2
SG168	TACGATA	2_R1	2_R2
SG170	ATGCAAT	2_R1	2_R2
T018	AGCTCCG	2_R1	2_R2
T019	AACTCG	2_R1	2_R2
T020	AGAATGCA	2_R1	2_R2
T021	GAATAGCA	2_R1	2_R2
T029	GCACCTCA	2_R1	2_R2
T041	ACGCT	1_R1	1_R2
T043	ATGGCG	1_R1	1_R2
T048	GGTCTT	2_R1	2_R2
T049	CAAGTAGA	2_R1	2_R2
T050	GCAAGAAT	2_R1	2_R2
T061	GGATTCA	1_R1	1_R2
T081	GCACCTCA	1_R1	1_R2
T082	ACTCCACG	1_R1	1_R2

T111	GCCAT	1_R1	1_R2
T112	TCATGG	1_R1	1_R2
T113	GCCTTA	1_R1	1_R2
T114	GATCCAA	1_R1	1_R2
T116	CACTGCCA	1_R1	1_R2
T118	TCACG	1_R1	1_R2
T119	CACGT	1_R1	1_R2
T130	CATCCG	1_R1	1_R2
T132	CTGGACA	1_R1	1_R2
T139	AGCTCCG	1_R1	1_R2
T142	CTGCA	1_R1	1_R2
T143	GTTCCA	1_R1	1_R2
T163	CCGTCA	1_R1	1_R2
T164	GATTACA	1_R1	1_R2
T167	AGACTCG	1_R1	1_R2
T169	CGCACACT	1_R1	1_R2
T170	TCCGCACA	1_R1	1_R2

References

Andersson A, Karlsson S, Ryman N, Laikre L (2022) 'Monitoring genetic diversity with new indicators applied to an alpine freshwater top predator' (Dryad).

Arnaud-Haond S, Alberto F, Eguiluz VM, Hernández-García E, Duarte CM, Serrão EA (2014) 'Data from: Disentangling the influence of mutation and migration in clonal seagrasses using the Genetic Distance Spectrum for microsatellites' (Dryad).

Benavente JN, Seeb LW, Seeb JE, Arismendi I, Hernández CE, Gajardo G, Galleguillos R, Cádiz MI, Musleh SS, Gomez-Uchida D (2016) 'Data from: Temporal genetic variance and propagule-driven genetic structure characterize naturalized rainbow trout (Oncorhynchus mykiss) from a Patagonian lake impacted by trout farming' (Dryad).

Buonomo R, Assis J, Fernandes F, Engelen AH, Airoldi L, Serrão EA (2016) 'Data from: Habitat continuity and stepping-stone oceanographic distances explain population genetic connectivity of the brown alga Cystoseira amentacea' (Dryad).

Burns KC, Berg J, Bialynicka-Birula A, Kratchmer S, Shortt K (2010) Tree diversity on islands: assembly rules, passive sampling and the theory of island biogeography. *Journal of Biogeography* **37**, 1876–1883.

Castro-Arellano I, Lacher TE, Willig MR, Rangel TF (2010) Assessment of assemblage-wide temporal niche segregation using null models. *Methods in Ecology and Evolution* **1**, 311–318.

CBD (2022) 'Draft decisions for the fifteenth meeting of the conference of the parties to the convention on biological diversity'.

Charbonnel E, Daguin C, Caradec L, Moittié E, Gilg O, Gavrilo M, Strom H, Mallory ML, Gilchrist G, Morrisson RIG, Leblois R, Roux C, Yearsley JM, Yannic G, Broquet T (2022) 'Searching for genetic evidence of demographic decline in an arctic seabird: beware of overlapping generations' (Dryad).

Clarke K, Warwick R 'Change in Marine Communities. An Approach to Statistical Analysis and Interpretation' (Primer-E Ltd: Plymouth, UK).

CLARKE KR, WARWICK RM (1998) A taxonomic distinctness index and its statistical properties. *Journal of Applied Ecology* **35**, 523–531.

Do C, Waples RS, Peel D, Macbeth GM, Tillett BJ, Ovenden JR (2014) NeEstimator v2: re-implementation of software for the estimation of contemporary effective population size (Ne) from genetic data. *Molecular ecology resources* **14**, 209–214.

Duplouy A, Wong SC, Corander J, Lehtonen R, Hanski I (2018) 'Data from: Genetic effects on life-history traits in the Glanville fritillary butterfly' (Dryad).

Excoffier L, Smouse PE, Quattro JM (1992) Analysis of molecular variance inferred from metric distances among DNA haplotypes: application to human mitochondrial DNA restriction data. *Genetics* **131**, 479–491.

Feijen F (2022) 'Molecular data and analysis specifications for a study on Atriophallophorus parasites from New Zealand' (Dryad).

Garner BA, Hoban S, Luikart G (2020) IUCN Red List and the value of integrating genetics. *Conservation Genetics* **21**, 795–801.

Gilbert KJ, Whitlock MC (2015) Evaluating methods for estimating local effective population size with and without migration. *Evolution; international journal of organic evolution* **69**, 2154–2166.

Gomez-Uchida D, Cañas-Rojas D, Riva-Rossi CM, Ciancio JE, Pascual MA, Ernst B, Aedo E, Musleh SS, Valenzuela-Aguayo F, Quinn TP, Seeb JE, Seeb LW (2019) 'Data from: Genetic signals of artificial and natural dispersal linked to colonization of South America by non-native Chinook salmon (Oncorhynchus tshawytscha)' (Dryad).

Gotelli NJ, Entsminger GL (2015) 'EcoSim: Null models software for ecology' (Zenodo).

Gotelli, Hart, Ellison (2015) 'EcoSimR: Null Model Analysis for Ecological Data' (Zenodo).

Goudet J, Jombart T (2022) 'hierfstat. Estimation and Tests of Hierarchical F-Statistics'.

Grünwald NJ, Goodwin SB, Milgroom MG, Fry WE (2003) Analysis of genotypic diversity data for populations of microorganisms. *Phytopathology* **93**, 738–746.

Grünwald NJ, Kamvar Z, Everhart SE, Tabima JF, Knaus BJ (2017) Population genetics and genomics in R.

Hamilton J, Gardner J, Piria M, Gavrilović A, Mrkonjić Fuka M, Svečnjak L, Nikolić S, Bakiu R (2023) 'Microsatellite data from Mediterranean mussels (Mytilus galloprovincialis) from the eastern coast of the Adriatic Sea' (Dryad).

Hess JE, Campbell NR, Docker MF, Baker C, Jackson A, Lampman R, McIlraith B, Moser ML, Statler DP, Young WP, Wildbill AJ, Narum SR (2014) 'Data from: Use of genotyping-by-sequencing data to develop a high-throughput and multi-functional SNP panel for conservation applications in Pacific lamprey' (Dryad).

Hoban S, Bruford MW, Funk WC, Galbusera P, Griffith MP, Grueber CE, Heuertz M, Hunter ME, Hvilsom C, Stroil BK, Kershaw F, Khoury CK, Laikre L, Lopes-Fernandes M, MacDonald AJ, Mergeay J, Meek M, Mittan C, Mukassabi TA, O'Brien D, Ogden R, Palma-Silva C, Ramakrishnan U, Segelbacher G, Shaw RE, Sjögren-Gulve P, Veličković N, Vernesi C (2021) Global

Commitments to Conserving and Monitoring Genetic Diversity Are Now Necessary and Feasible. *Bioscience* **71**, 964–976.

Jost L (2008) G(ST) and its relatives do not measure differentiation. *Molecular ecology* **17**, 4015–4026.

Kamvar ZN, Tabima JF, Grünwald NJ (2014) Poppr: an R package for genetic analysis of populations with clonal, partially clonal, and/or sexual reproduction. *PeerJ* **2**, e281.

Kort H de, Vandepitte K, Mergeay J, Mijnsbrugge KV, Honnay O (2015) 'Data from: The population genomic signature of environmental selection in the widespread insect-pollinated tree species Frangula alnus at different geographical scales' (Dryad).

Krehenwinkel H, Tautz D (2013) 'Data from: Northern range expansion of European populations of the wasp spider Argiope bruennichi is associated with global warming correlated genetic admixture and specific temperature adaptations' (Dryad).

Lehnert SJ, DiBacco C, Jeffery NW, Blakeslee AM, Isaksson J, Roman J, Wringe BF, Stanley RR, Matheson K, McKenzie CH, Hamilton LC, Bradbury IR, Stanley RRE, Blakeslee AMH (2018) 'Data from: Temporal dynamics of the genetic clines of invasive European green crab (Carcinus maenas) in eastern North America' (Dryad).

Major EI, Höhn M, Avanzi C, Fady B, Heer K, Opgenoorth L, Piotti A, Popescu F, Postolache D, Vendramin GG, Csilléry K (2021) 'Data from: Fine-scale spatial genetic structure across the species range reflects recent colonization of high elevation habitats in silver fir (Abies alba Mill.)' (Dryad).

Marandel F, Charrier G, Lamy J-B, Le Cam S, Lorance P, Trenkel VM (2020) Estimating effective population size using RADseq: Effects of SNP selection and sample size. *Ecology and evolution* **10**, 1929–1937.

McLaughlin CM, Hinshaw C, Sandoval-Arango S, Zavala-Paez M, Hamilton JA (2025) Redlisting genetics: towards inclusion of genetic data in IUCN Red List assessments. *Conservation Genetics* **26**, 213–223.

Mesnick SL, Taylor BL, Archer FI, Martien KK, Escorza Treviño S, Hancock-Hanser BL, Moreno Medina SC, Pease VL, Robertson KM, Straley JM, Baird RW, Calambokidis J, Schorr GS, Wade P, Burkanov V, Lunsford CR, Rendell L, Morin PA (2010) 'Data from: Sperm whale population structure in the eastern and central North Pacific inferred by the use of single nucleotide polymorphisms (SNPs), microsatellites and mitochondrial DNA' (Dryad).

Miller SM, Harper CK, Bloomer P, Hofmeyr J, Funston PJ (2014) 'Data from: Evaluation of microsatellite markers for populations studies and forensic identification of African lions (Panthera leo)' (Dryad).

Nakanishi A (2024) 'Microsatellite genotypes of Cercidiphyllum japonicum seeds and identity numbers of the seed parents' (Dryad).

Nygaard M, Kopatz A, Speed JM, Martin MD, Prestø T, Kleven O, Bendiksby M (2022) 'Spatiotemporal monitoring of the rare Northern dragonhead, Dracocephalum ruyschiana (Lamiaceae): SNP genotyping and environmental niche modelling herbarium specimens' (Dryad).

Oksanen J, Simpson G, Blanchet F et al. (2022) 'vegan. Community Ecology Package'.

Olah G, Stojanovic D, Webb MH, Waples RS, Heinsohn R (2021) Comparison of three techniques for genetic estimation of effective population size in a critically endangered parrot. *Animal Conservation* **24**, 491–498.

Pertoldi C (2022) 'Strong isolation by distance among local populations of an endangered butterfly species (Euphydryas aurinia)' (Dryad).

Prasetyo E, Widiyatno W, Indrioko S, Na'iem M, Matsui T, Matsuo A, Suyama Y, Tsumura Y (2020) 'Genetic diversity and the origin of commercial plantation of Indonesian teak on Java Island' (Dryad).

Quesada-Calderon S, Giles EC, Morales-González S, Saenz-Agudelo P (2021) 'Pinpointing genetic breaks in the southeastern Pacific: phylogeography and genetic structure of Pyura chilensis, a commercially important tunicate' (Dryad).

Roberts JMK, Anderson DL, Tay WT (2015) 'Data from: Multiple host-shifts by the emerging honeybee parasite, Varroa jacobsoni' (Dryad).

Robinson Z (2019) 'RLDNe. A Convenient R Interface For NeEstimator'.

Ruiz Daniels R, Taylor RS, Serra-Varela MJ, Vendramin GG, González-Martínez SC, Grivet D (2018) 'Data from: Inferring selection in instances of long-range colonization: the Aleppo pine (Pinus halepensis) in the Mediterranean Basin' (Dryad).

Schweiger O, Klotz S, Durka W, Kühn I (2008) A comparative test of phylogenetic diversity indices. *Oecologia* **157**, 485–495.

Shimatani K (2001) On the measurement of species diversity incorporating species differences. *Oikos* **93**, 135–147.

SIMPSON EH (1949) Measurement of Diversity. *Nature* **163**, 688.

Sun ZX, Zhai YF, Zhang JQ, Kang K, Cai JH, Fu Y, Qiu JQ, Shen JW, Zhang WQ (2015) 'Data from: The genetic basis of population fecundity prediction across multiple field populations of Nilaparvata lugens' (Dryad).

Therkildsen NO, Hemmer-Hansen J, Hedeholm RB, Wisz MS, Meldrup D, Bonanomi S, Retzel A, Olsen SM, Nielsen EE, Pampoulie C (2015) 'Data from: Spatiotemporal SNP analysis reveals pronounced biocomplexity at the northern range margin of Atlantic cod Gadus morhua' (Dryad).

Triest L (2021) 'Microsatellite data of Avicennia marina from Gazi Bay, Kenya' (Dryad).

Valade R, Kenis M, Hernandez-Lopez A, Augustin S, Mena NM, Magnoux E, Rougerie R, Lakatos F, Roques A, Lopez-Vaamonde C (2010) 'Data from: Mitochondrial and microsatellite DNA markers reveal a Balkanic origin for the highly invasive Horse-Chestnut leaf miner Cameraria ohridella (Lepidoptera, Gracillariidae)' (Dryad).

van Hooft P, Getz W, Greyling B, Heller R, Røed K, Bastos A (2021) 'Microsatellite data from various African buffalo (Syncerus caffer) populations throughout Africa' (Dryad).

Wendt A, Haas C, Gorman T, Roberts J (2021) 'Larval A. bishopi microsatellite data from: Metapopulation genetics of endangered reticulated flatwoods salamanders (Ambystoma bishopi) in a dynamic and fragmented landscape' (Dryad).

Zachos FE, Frantz AC, Kuehn R, Bertouille S, Colyn M, Niedziałkowska M, Pérez-González J, Skog A, Sprĕm N, Flamand M-C (2016) Genetic Structure and Effective Population Sizes in European

Red Deer (Cervus elaphus) at a Continental Scale: Insights from Microsatellite DNA. *The Journal of heredity* **107**, 318–326.
